# Decompression alone versus decompression with instrumented fusion in the treatment of lumbar degenerative spondylolisthesis: a systematic review and meta-analysis of randomised trials

**DOI:** 10.1136/jnnp-2022-330158

**Published:** 2023-02-27

**Authors:** Radek Kaiser, Lucia Kantorová, Alena Langaufová, Simona Slezáková, Dagmar Tučková, Miloslav Klugar, Zdeněk Klézl, Pavel Barsa, Jan Cienciala, Richard Hajdúk, Lumír Hrabálek, Roman Kučera, David Netuka, Martin Prýmek, Martin Repko, Martin Smrčka, Jan Štulík

**Affiliations:** 1 Department of Neurosurgery and Neurooncology, First Faculty of Medicine, Charles University, Prague, Czech Republic; 2 Military University Hospital Prague, Prague, Czech Republic; 3 Czech National Centre for Evidence-Based Healthcare and Knowledge Translation (Cochrane Czech Republic, Czech EBHC: JBI Centre of Excellence, Masaryk University GRADE Centre), Institute of Biostatistics and Analyses, Masaryk University Faculty of Medicine, Brno, Czech Republic; 4 Czech Health Research Council, Prague, Czech Republic; 5 Institute of Health Information and Statistics of the Czech Republic, Prague, Czech Republic; 6 Department of Spinal Surgery, First Faculty of Medicine, Charles University, Prague, Czech Republic; 7 Motol University Hospital, Prague, Czech Republic; 8 Department of Neurosurgery, Regional Hospital Liberec, Liberec, Czech Republic; 9 Department of Orthopaedic Surgery, Faculty of Medicine, Masaryk University, Brno, Czech Republic; 10 University Hospital Brno, Brno, Czech Republic; 11 Department of Neurosurgery, Faculty of Medicine and Dentistry, Palacky University, Olomouc, Czech Republic; 12 University Hospital Olomouc, Olomouc, Czech Republic; 13 Department of Neurosurgery, Na Homolce Hospital, Prague, Czech Republic; 14 Department of Neurosurgery, Faculty of Medicine, Masaryk University, Brno, Czech Republic

## Abstract

**Objective:**

To determine the efficacy of adding instrumented spinal fusion to decompression to treat degenerative spondylolisthesis (DS).

**Design:**

Systematic review with meta-analysis.

**Data sources:**

MEDLINE, Embase, Emcare, Cochrane Library, CINAHL, Scopus, ProQuest Dissertations & Theses Global, ClinicalTrials.gov and WHO International Clinical Trials Registry Platform from inception to May 2022.

**Eligibility criteria for study selection:**

Randomised controlled trials (RCTs) comparing decompression with instrumented fusion to decompression alone in patients with DS. Two reviewers independently screened the studies, assessed the risk of bias and extracted data. We provide the Grading of Recommendations, Assessment, Development and Evaluation assessment of the certainty of evidence (COE).

**Results:**

We identified 4514 records and included four trials with 523 participants. At a 2-year follow-up, adding fusion to decompression likely results in trivial difference in the Oswestry Disability Index (range 0–100, with higher values indicating greater impairment) with mean difference (MD) 0.86 (95% CI −4.53 to 6.26; moderate COE). Similar results were observed for back and leg pain measured on a scale of 0 to 100, with higher values indicating more severe pain. There was a slightly increased improvement in back pain (2-year follow-up) in the group without fusion shown by MD −5·92 points (95% CI −11.00 to −0.84; moderate COE). There was a trivial difference in leg pain between the groups, slightly favouring the one without fusion, with MD −1.25 points (95% CI −6.71 to 4.21; moderate COE). Our findings at 2-year follow-up suggest that omitting fusion may increase the reoperation rate slightly (OR 1.23; 0.70 to 2.17; low COE).

**Conclusions:**

Evidence suggests no benefits of adding instrumented fusion to decompression for treating DS. Isolated decompression seems sufficient for most patients. Further RCTs assessing spondylolisthesis stability are needed to determine which patients would benefit from fusion.

**PROSPERO registration number:**

CRD42022308267.

**WHAT IS ALREADY KNOWN ON THIS TOPIC**

- Survey studies show significant heterogeneity among spine surgeons in the surgical management of degenerative spondylolisthesis. Although some systematic reviews have reported higher efficacy of decompression with fusion compared with isolated decompression, other reviews failed to find significant differences between the interventions. Based on available evidence, professional spine surgery societies recommend isolated decompression in cases without signs of instability. However, previously published meta-analyses inappropriately combined randomised trials with observational studies and did not provide a Grading of Recommendations, Assessment, Development and Evaluation (GRADE) and assessment of the certainty of evidence. Due to conflicting conclusions from previous studies, degenerative spondylolisthesis is still the most common indication for elective spinal fusion with an increasing trend and high costs.

**WHAT THIS STUDY ADDS**

- Our systematic review with meta-analysis is the broadest and most robust analysis in the field. The review design was discussed and validated by a national panel of experts in spine surgery with extensive experience in treating degenerative spinal conditions and evidence synthesis experts. We included trials using pedicle screw fixation fusion and excluded those using other techniques of fixation or non-instrumented fusion and trials with pseudo-randomisation. Expert information specialists devised complex search strategies and searched nine sources in May 2022. We also provide the GRADE Summary of Findings.

**HOW THIS STUDY MIGHT AFFECT RESEARCH, PRACTICE OR POLICY**

- The evidence suggests that adding fusion to decompression is ineffective for most patients with spondylolisthesis. It likely results in no additional benefits regarding disability, pain and quality of life at a minimum follow-up of 2 years after surgery. Furthermore, fusion is linked to increased surgery-related complications (duration, blood loss, extended hospital stay) and costs while not decreasing the long-term complication and reoperation rate. Evidence from randomised trials is missing to compare stable versus unstable spondylolisthesis, and future trials should aim to determine which subgroups would benefit from adding fusion to decompression.

## Introduction

Degenerative spondylolisthesis (DS) is a widespread spinal pathology with prevalence reaching 25%–43% in women and 19%–31% in men over 65 years.^1^ Ventral shift of the cranial vertebra compared with the more caudal vertebra is caused by arthritis of the facet joints, malfunction of the stabilising ligaments and disc degeneration. All these changes contribute to the compromise of the canal and lumbar spinal stenosis (LSS). DS is one of the most common causes of progressive lower back or leg pain (neurogenic claudication or radiculopathy). It is a common indication for spinal surgery in adults,^2^ generally leading to better results than conservative therapy.^3 4^

The least invasive, safest and least costly procedure to treat DS is non-destabilising decompression of the spinal canal with resection of hypertrophic facet joints and ligamentum flavum.^5^ There is an ongoing debate on whether fusion of the altered lumbar segment should be added to decompression to decrease the risk of further progression of the pathology. Although some reviews have found greater efficacy of fusion in the treatment of DS,^6 7^ other studies have not unequivocally demonstrated the advantage of fusion.^5 8^ However, DS represents the most common indication for elective spinal fusion with a highly increasing tendency and high hospital costs.^9 10^

In February 2022, we searched Epistemonikos, MEDLINE (via Ovid) and PROSPERO for any ongoing or completed reviews. We found seven completed systematic reviews^5–8 11–13^ and four registered reviews (all completed and retrieved, see online supplemental appendix E). Since then, another systematic review has been published.^14^ However, most searched only for English-language studies, included studies with outdated and no longer used fusion techniques and inappropriately^15^ combined the results of randomised controlled trials (RCTs) and pseudo-RCTs or observational studies in meta-analyses. None provided the Grading of Recommendations, Assessment, Development and Evaluation (GRADE) assessment of certainty of evidence (COE).

10.1136/jnnp-2022-330158.supp1 Supplementary data

Therefore, we did a systematic review and meta-analysis of RCTs to compare decompression alone and decompression with instrumented fusion in treating DS across relevant outcomes and prespecified subgroups of patients.

## Methods

This report is a systematic review with meta-analysis driven by the following question: What is the effectiveness and safety of decompression with instrumented fusion versus decompression only in degenerative lumbar spondylolisthesis? The review followed a priori-developed protocol, the Cochrane Handbook,^15^ and the Preferred Reporting Items for Systematic Reviews and Meta-Analyses reporting guidelines.^16^ We interpreted the findings as suggested by Santesso *et al*.^17^ Selection criteria for included studies are shown in table 1.

**Table 1:** Selection criteria

Population	Adults with degenerative lumbar spondylolisthesis (excluded: patients with isthmic spondylolisthesis, degenerative scoliosis, spinal stenosis with other causes, or those with previous spinal surgery)
Intervention	Isolated decompression (any surgical technique)
Comparison	Decompression with fusion (instrumented spinal fusion with pedicle screw fixation)
Outcomes	**Primary**: **Oswestry Disability Index** (scale 0–100, with higher scores indicating more severe impairment) **Low back pain and leg pain** (on a visual analogue scale (VAS) of 0–100, with higher scores indicating more pain) **Secondary**: reoperation rate, complication rate, length of hospital stay, duration of surgery, blood loss during surgery, and quality of life (QoL)
Studies	Randomised controlled trials with true randomisation (pseudo-RCTs excluded), any language or location

RCT, randomised controlled trial.

### Search strategy and selection criteria

The search strategy was modelled based on the North American Spine Society, Diagnosis and Treatment of Degenerative Lumbar Spondylolisthesis,^18^ modified by information specialists and validated by clinical experts and an evidence synthesis expert. A three-step search strategy was used to identify both published and unpublished studies. A limited search was run in MEDLINE (Ovid) and EMBASE (Ovid), using the keywords and index terms ‘lumbar spondylolisthesis’ and ‘fusion’. The analysis of the text words in the title and abstract and the index terms used to describe articles followed.

In May 2022, an information specialist conducted a comprehensive search including MEDLINE (Ovid), Embase (Ovid), Emcare (Ovid), Cochrane Library, CINAHL (EBSCO) and Scopus. Sources of grey literature included ProQuest Dissertations & Theses Global and registers of clinical trials: ClinicalTrials.gov and WHO International Clinical Trials Registry Platform (see online supplemental appendix A). The reference lists of relevant publications were screened for additional eligible studies. We did not apply time, study design, language, geographical or other restrictions. We imported the retrieved records into Endnote V.X9.2 (Clarivate Analytics, Pennsylvania). We removed the duplicates according to the method described by Bramer *et al*.^19^ Search strategies were downloaded/manually copied from each database or register and saved in a Microsoft Word document.

Two reviewers (LK and DT) independently screened the titles and abstracts and the full texts of the potentially eligible records. We resolved any conflicts by discussion and with a third reviewer (RKa). We recorded and reported the reasons for excluding records at full-text screening (online supplemental appendix C). We contacted the authors for any missing information.

We included RCTs comparing isolated decompression (any surgical technique) with decompression with fusion in adult participants with DS with at least 12 months of follow-up. Only trials with instrumented spinal fusion with pedicle screw fixation were included. Excluded were cases with isthmic spondylolisthesis, degenerative scoliosis, spinal stenosis with other causes or those with previous spinal surgery. Primary outcomes were the Oswestry Disability Index (ODI), back pain and leg pain. Secondary outcomes were reoperation rate, complication rate, length of hospital stay, duration of surgery, blood loss during surgery and quality of life (QoL). No institutional review board approval was required for this meta-analysis because the study included previously published data.

### Data extraction

Two reviewers (LK and DT) independently extracted data on the characteristics of the eligible studies using our data extraction form: author, publication year, title, country, inclusion and exclusion criteria, sample sizes per protocol, at randomisation/allocation and interventions received, characteristics of the interventions, length of follow-up and baseline data of the samples, which included age, sex, severity and type of spondylolisthesis. We then extracted data on outcome measurements (see above), preferring a 2-year follow-up. All steps of the review process were discussed and validated by the national panel of experts in spine surgery consisting of six neurosurgeons and six orthopaedic surgeons.

### Quality assessment

Two reviewers (LK and DT) separately assessed study quality using the Cochrane risk-of-bias tool for randomised trials (RoB 2).^20^ Any disagreements were resolved by discussion or with a third reviewer (MK). The results are shown in online supplemental appendix B using the *robvis* tool^21^ to visualise the risk of bias.

### Data analysis

We undertook statistical pooling for all outcomes where at least two study results were available via Cochrane RevMan software (Review Manager (RevMan) (Computer program), V.5.4, The Cochrane Collaboration, 2020), if appropriate. We pooled data with a minimum follow-up of 2 years (only relevant for ODI, pain and reoperation rate). If the 2-year follow-up data were unavailable, we chose data for a longer follow-up of up to 5 years. If the CI or SE were available, we calculated the SD using the software tools. We transformed the values for pain measured on a numeric rating scale from 0 to 10 to a visual analogue scale (VAS) ranging from 0 to 100. We used the VAS scale throughout the review.

We presented the summary estimate as an OR with 95% CI using the Mantel-Haenszel random effects model for the dichotomous data. We estimated the OR using intention-to-treat data, if available. The dichotomous data included the complication and reoperation rate.

We presented the mean difference (MD) with 95% CI for the continuous data using the Mantel-Haenszel random effects model. The continuous data included the outcomes of ODI, leg pain, back pain, blood loss during surgery, duration of the surgery and length of hospitalisation.

We planned subgroup analyses for categories of DS (specifically, according to stability); however, data were unavailable to allow statistical pooling.

### Rating the certainty of evidence

The GRADE approach for grading the COE was followed, and a Summary of Findings (SoF) was created using GRADEPro GDT (McMaster University, ON, Canada).^22^ The SoF presents the following information where appropriate: absolute risks for treatment and control, estimates of relative risk and a ranking of COE based on the risk of bias, directness, heterogeneity, precision and risk of publication bias of the review results. To determine the risk of publication bias, we used general indicators (eg, geographical distribution of the studies), as there were too few studies to use a statistical tool for analysis. We assessed statistical between-trial heterogeneity using the I^2^ statistics.^15^ We used the minimal clinically important differences determinations proposed by Asher *et al* to interpret the results.^23^

### Patient and public involvement

This study is a meta-analysis of previously published studies. No patients were involved in setting the research question or the outcome measures, nor were they involved in developing plans for the design and conduct of the study. No patients were asked to advise on interpretation or writing up of results.

## Results

**Figure 1:**
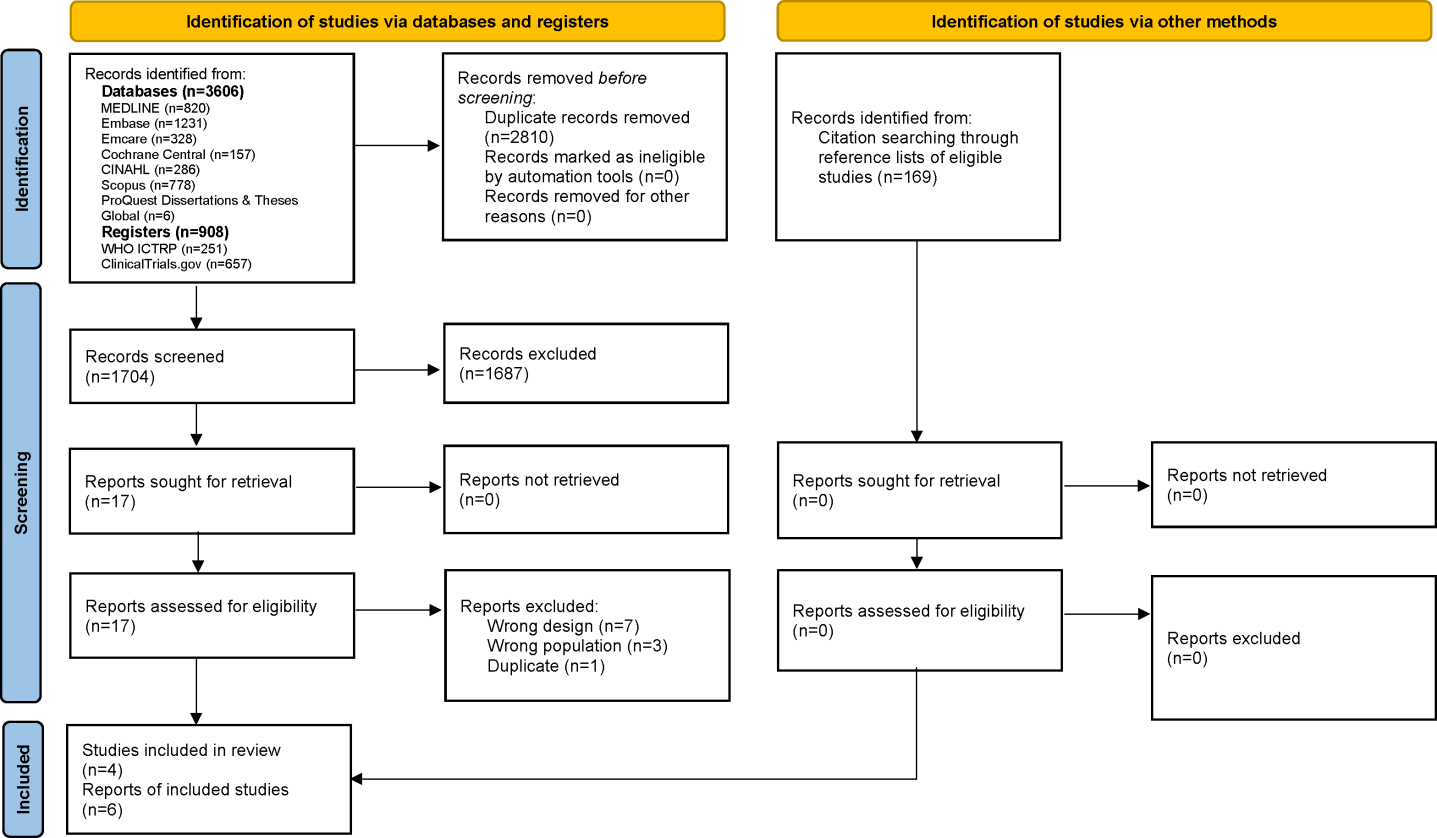
Study selection.

Our systematic search gave 4514 records. After removing duplicates, we screened 1704 records identified through database searching and 169 through reference list searching and assessed the full texts of 17 reports against the eligibility criteria for this review. We excluded 11 reports that did not meet the criteria or did not use proper randomisation (online supplemental appendix C). Finally, we included four RCTs (six reports).^24–29^ See figure 1 for an overview of the search and screening process.

The four included trials published between 2016 and 2021 with 60^26^ to 262^24^ participants randomly allocated LSS patients with DS either to decompression only or decompression with instrumented fusion. Försth *et al* did not measure instability, Ghogawala *et al* included only stable spondylolisthesis patients and Austevoll and Inose *et al* had 21%–26% and 41%–42% patients with instability defined as ≥3 mm forward translation or ≥10°C of angular rotation, respectively, assessed on dynamic standing radiographs. All studies provided a minimum of 24 months of follow-up, with Inose providing 1 and 5-year follow-ups and recently making data available for a 12-year follow-up.^25^ The mean age of patients spanned 61–68 years across the four trials. The mean degree of vertebral slip ranged from 5.6 mm to 8.1 mm. Table 2 presents the complete characteristics of the included trials (for details, see online supplemental appendix F).

**Table 2:** Characteristics of the included randomised controlled trials and baseline data

Author, year country	Participants	Definition of DS	Surgical approach	Outcomes	Risk of bias domains	FU (months)	Interventions	Sample size	Age (years), mean (SD)	Gender (female), n (%)	Degree of vertebral slip (mm), mean (SD)	Dynamic instability, n (%)	VAS-back, mean (SD)	VAS-leg, mean (SD)
Austevoll, et al 2021 NORDSTEN-DS trial Norway^24^	18–80 years; neurogenic claudication or radicular radiating pain in the lower limbs that had not responded to at least 3 months of conservative care; LSS; DS≥3 mm Mix of stable and instable	Dynamic instability—slippage of at least 3 mm, or at least 10 degrees of angulation, as assessed by dynamic standing radiographs	Posterior decompression (with or without preservation of midline structures) was used, followed by implantation of pedicle screws with rods and bone grafting across the level of spondylolisthesis, with optional use of an intervertebral fusion device	ODI, ZCQ, NRS back and leg, EQ-5D-3L, duration of surgery and length of hospital Stay, complications and reoperations and the percentage of patients who responded that their condition was ‘much worse’ or ‘worse than ever’ on the Global Perceived Effect scale	True randomisation of 1:1 ratio Allocation: computer-generated, stratified according to centre in blocks of 4–6 Blinding: patients likely not blinded; Investigators (outcome assessment) blinded Modified intention-to-treat analysis: all patients who received the trial treatment in accordance with the randomisation with available data (with imputations for missing data)	3, 24	D	133	66.0±7.4	92 (69.2)	7.6 (3.2)	35 (26)	6.7±2.0	6.6±2.0
							DF	129	66.5±7.9	88 (68.2)	7.2 (2.8)	27 (21)	6.7±2.1	6.7±2.1
Försth, et al 2016 Sweden^28^	LSS with or without DS (only those with DS used in this review); 50 and 80 years Mix of stable and instable (not measured)	Conventional lateral radiography (flexion–extension radiographs were not obtained); forward slip ≥3 mm	Method determined by surgeon	ODI, EQ-5D, VAS back and leg, ZCQ, patient reported outcomes (satisfaction), 6 min walk test		24 and 60	D	68	67 (7)	56 (82.4)	7.4 (2.8)	–	6.3 (2.4)	6.5 (2.2)
							DF	67	68 (7)	51 (76.1)	7.4 (2.6)	–	6.4 (2.0)	6.4 (2.1)
Ghogawala, et al 2016 SLIP trial USA^27^	DS, grade I, LSS and neurogenic claudication with or without lumbar radiculopathy; **only stable** (motion≤3 mm)	3–14 mm (mean 1.3 and 1.6)	Posterolateral instrumented fusion (a lumbar laminectomy as well as implantation of pedicle screws and titanium alloy rods across the level of listhesis, with a bone graft harvested from the iliac crest)	SF-36 physical component, ODI, complications and reoperations, blood loss, operative time, and length of stay		12, 24 and 48	D	35	66.5 (8.0)	27 (77)	6.5 (2.3)	–	–	–
							DF	31	66.7 (7.2)	26 (84)	5.6 (2.2)	–	–	–
Inose, et al 2018 Japan^26^	One level LSS with DS at the L4/5 level Mix of stable and instable	>3 mm of spondylolisthesis of the L4 vertebra on a plain lateral radiograph Dynamic instability—change of >10 degrees of angulation or >4 mm of translation of the vertebrae between flexion and extension of the spine	Posterolateral fusion with autogenous iliac bone graft and pedicle screw fixation	JOA, VAS back and leg, duration of operation, blood loss, duration of postoperative hospital stay and major intraoperative and perioperative complications, degree of progression of slippage (>5%) at postoperative year 5		12, 60, 144	D	29	63.4 (8.6)	12 (41)	6.5 (2.2)	12 (41)	5.28 (3.11)*	6.21 (2.3)*
							DF	31	61.2 (6.7)	20 (65)	8.1 (3.8)	13 (42)	6.28 (3.01)*	7.68 (2.51)*

*Unpublished data from authors.

D, decompression only; DF, decompression with fusion; DS, degenerative spondylolisthesis; EQ-5D, European Quality of Life–5 Dimensions; FU, follow-up; JOA, Japanese Orthopaedic Association score; LSS, lumbar spinal stenosis; NRS, numeric rating scale; ODI, Oswestry Disability Index; SF-36, Short Form 36 Health Survey Questionnaire; VAS, visual analogue scale; ZCQ, Zurich Claudication Questionnaire.

**Figure 2:**
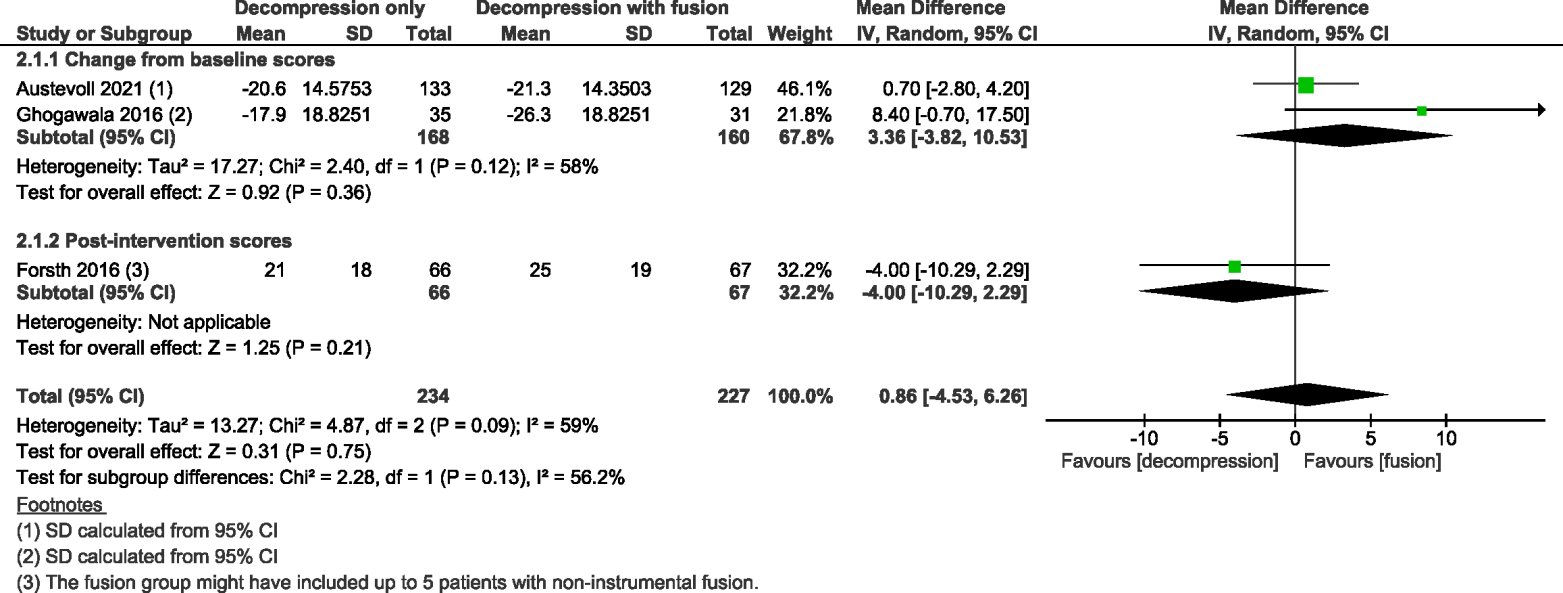
Forest plot for the Oswestry Disability Index (ODI, measured on a scale of 0–100, where 100 indicates the most severe disability) at the 2-year follow-up. ‘Favours fusion’ means the ODI was lower (or improved more) in the decompression+fusion group.

**Figure 3:**
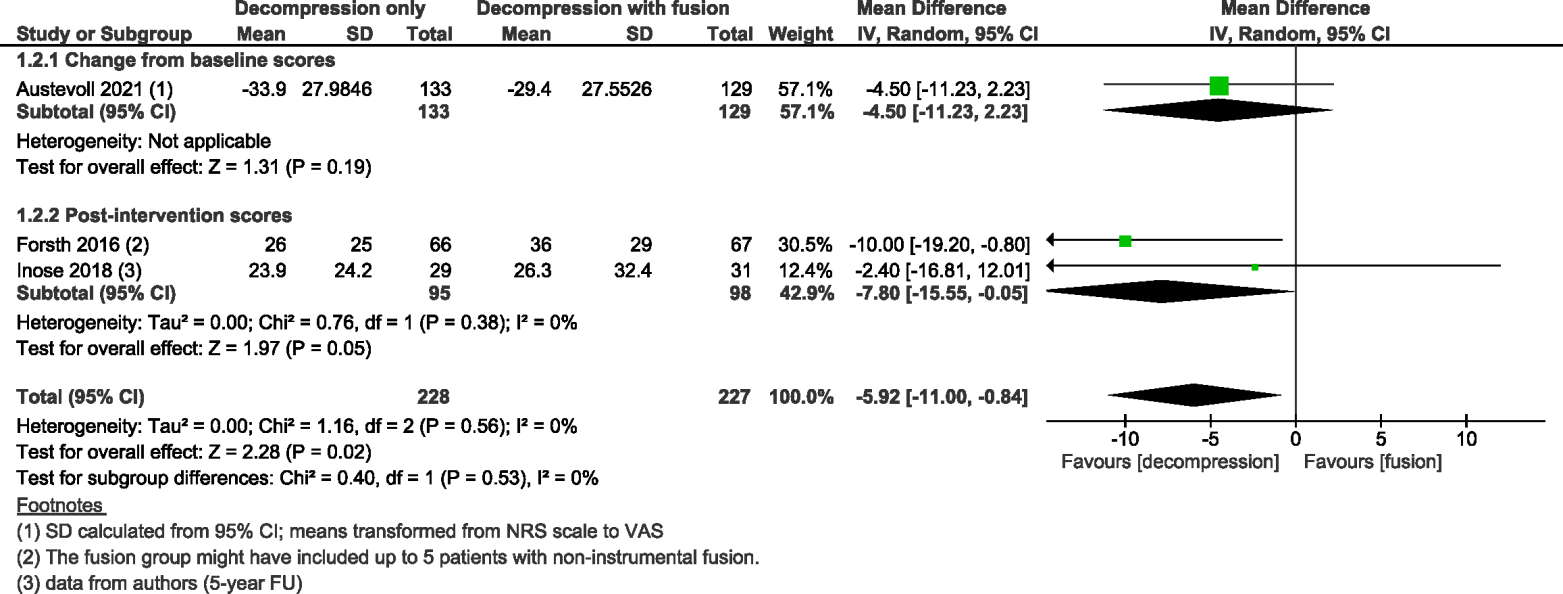
Forest plot for back pain (measured on a scale from 0 to 100, with 100 indicating the most severe pain) at a minimal follow-up of 2 years. ‘Favours fusion’ means the back pain score was lower (or improved more) in the decompression+fusion group.

**Figure 4:**
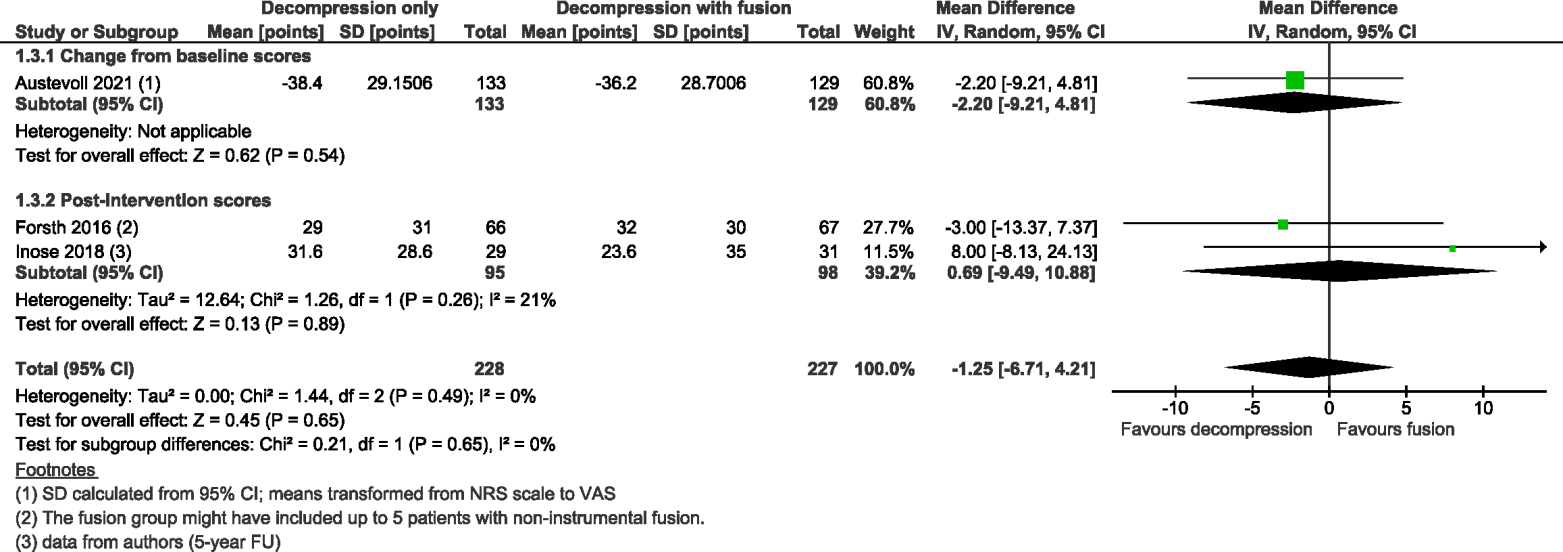
Forest plot for leg pain (measured on a scale from 0 to 100, where 100 indicates the most severe pain) at a minimal follow-up of 2 years. ‘Favours fusion’ means the leg pain score was lower (or improved more) in the decompression+fusion group.

Three trials^24 27 28^ with 461 participants suggested adding fusion to decompression likely results in little to no difference in ODI at 2-year follow-up with MD 0·86 (as measured on a scale of 0–100, with 100 indicating more severe impairment; 95% CI from −4·53 to 6·26; I^2^=59%; moderate COE, figure 2), slightly favouring the group with fusion. Three trials^24 26 28^ with 455 participants showed decompression without fusion likely reduces back pain slightly more compared with the group that underwent decompression with fusion, with MD −5·92 (scale 0–100, with higher numbers indicating more pain; 95% CI from −11·00 to −0·84; I^2^=0%; moderate COE, figure 3) and results in little to no difference between the two groups in leg pain with MD −1·25 (95% CI from −6·71 to 4·21; I^2^=0%; moderate COE, figure 4); however, slightly favouring the group without fusion.

Three trials^24 26 28^ with 436 participants showed that both interventions might result in a similar reoperation rate with OR 1·23 (95% CI from 0·70 to 2·17; I^2^=0%; low COE, online supplemental figure S1), slightly favouring the group with fusion. Three trials^24 26 27^ with 388 participants showed 30 complications in the decompression only group and 56 complications in the fusion group (low COE, see online supplemental appendix DAppendix D). The same trials showed that decompression without fusion likely results in a considerable reduction in the length of hospital stay with MD −1·7 days (95% CI from −1·75 to −1·65; I^2^=0%; moderate COE, online supplemental figure S2). Four trials^24 26–28^ with 521 participants showed that decompression without fusion likely results in a shorter surgery with MD −93·97 mins (95% CI from −125·44 to −62·50; I^2^=95%; moderate COE, online supplemental figure S3). The same trials also showed blood loss is probably greatly reduced when fusion is not performed with MD −320·55 mL (95% CI from −389·61 to −251·49; I^2^=60%; moderate COE; online supplemental figure S4). No meaningful differences were identified between the two groups in QoL.

The risk of outcome bias across all studies was similar and predominately ‘low’. The authors poorly reported the details of blinding patients and outcome assessors; however, blinding may be difficult for these interventions and its absence may not significantly impact the results. Therefore, we assessed the risk of bias as of ‘some concern.’ Table 3 shows the GRADE assessments of the COE. COE started at high and was further downgraded for all outcomes by one or two levels. We downgraded for risk of bias (inadequate blinding in subjectively assessed outcomes) and imprecision (low number of events and wide CIs). Some heterogeneity was identified, but for surgery-related outcomes, it could be explained by the inherent differences in workplaces and the decompression method chosen. We downgraded for unexplained heterogeneity for complication rate and ODI. The final COE was low to moderate across outcomes.

**Table 3:** The GRADE summary of findings table

Outcomes	Number of participant (studies) follow-up	Certainty of the evidence (GRADE)	Relative effect (95% CI)	Anticipated absolute effects‡‡	
				Risk with decompression and fusion	Risk difference with decompression
ODI (FU 2 years) Scale: 0–100 Higher ODI indicates more impairment	461 (3 RCTs)	⨁⨁⨁〇 Moderate*	**MD 0.86** (−4.53 to 6.26)	–	MD **0.86 points higher** (4.53 lower to 6.26 higher)
Back pain (FU 2 years) Scale: 0–100 Higher number indicates more pain	455 (3 RCTs)	⨁⨁⨁〇 Moderate†	**MD −5.92** (−11.00 to −0.84)	–	MD **5.92 points lower** (11 lower to 0.84 lower)
Leg pain (FU 2 years) Scale: 0–100 Higher number indicates more pain	455 (3 RCTs)	⨁⨁⨁〇 Moderate†	**MD −1.25** (−6.71 to 4.21)	–	MD **1.25 points lower** (6.71 lower to 4.21 higher)
Reoperation rate (FU 2 years)	436 (3 RCTs)	⨁⨁〇〇 Low‡§	**OR 1.23** (0.70 to 2.17)	128 per 1 000	**25 more per 1 000** (35 fewer to 113 more)
Complication rate (intraoperative and perioperative complications, up to 3 months after discharge)	388 (3 RCTs)	⨁⨁〇〇 Low¶	There were 30 complications in the decompression only group and 56 complications in the fusion group, with data driven mainly by Austevoll *et al* due to the highest number of recorded complications. When counting only the complications that occurred during hospital stay in Austevoll trial, the total number of complications in the three trials is 20 and 38.		
Length of hospital stay (days)	388 (3 RCTs)	⨁⨁⨁〇 Moderate‡	**MD −1.70** (−1.75 to −1.65)	–	MD **1.7 days lower** (1.75 lower to 1.65 lower)
Duration of surgery (min)	521 (4 RCTs)	⨁⨁⨁〇 Moderate**	**MD −93.97** (−125.44 to −62.50)	–	MD **93.97 mins shorter** (125.44 lower to 62.5 lower)
Blood loss during surgery (mL)	521 (4 RCTs)	⨁⨁⨁〇 Moderate††	**MD −320.55** (−389.61 to −251.49)	–	MD **320.55 mL lower** (389.61 lower to 251.49 lower)
Quality of life	397 (2 RCTs)	⨁⨁⨁〇 Moderate§	In Forsth *et al,* the quality of life was measured by EQ-5D with the decompression only group (n=66) having a 2y FU score of 0.69±0.28 and the decompression plus fusion group score 0.63±0.31 (p=0.20). (Scale −0.59 to 1.0, Higher score=better quality of life). Austevoll *et al* used the same scale to assess QoL with decompression-alone group showing a score of 0.70 (95% CI 0.65 to 0.75) at 2-year follow-up and fusion group 0.72 (95% CI 0.67 to 0.76). No important differences were identified between the groups in quality of life.		

We used the following minimal clinically important differences: ODI 14·3, back pain 16, leg pain 17 points.

*Downgraded by one level due to heterogeneity of 59%.

†Downgraded by one level due to imprecision for low number of events.

‡Downgraded by one level for risk of bias (lack of blinding).

§Downgraded by one level for imprecision.

¶Downgraded by two levels for concerns with risk of bias (inadequate blinding), high imprecision due to low number of events, and small heterogeneity between trials.

**Downgraded by one level for risk of bias. Heterogeneity was high (95%), however, it can be explained by the character of the outcome and the differences in the method of measurement, practices, skills and the operational methods at the respective workplaces.

††Downgraded by one level for risk of bias. Heterogeneity was high (60%), however, it can be explained by the differences in measurement of the outcome across workplaces (trials) and the chosen methods of decompression. The results of trials, however, were consistently in favour of decompression only and all clinically significant. We, therefore, did not downgrade for inconsistency.

‡‡The risk in the intervention group (and its 95% CI) is based on the assumed risk in the comparison group and the relative effect of the intervention (and its 95% CI).

EQ-5D, European Quality of Life–5 Dimensions; FU, follow-up; GRADE, Grading of Recommendations, Assessment, Development and Evaluation; MD, mean difference; ODI, Oswestry Disability Index; QoL, quality of life; RCT, randomised controlled trial.

## Discussion

This review shows that spinal fusion may not be necessary in most cases of DS. At 2 years after surgery, the results for ODI, leg pain, reoperation rate and QoL are comparable between both groups (decompression with and without fusion), and omitting fusion likely reduces back pain slightly more compared with decompression with fusion. Isolated decompression is linked with fewer perioperative complications. Fusion is associated with a notable increase in the duration of surgery, blood loss and extended hospital stay. Data were not available to assess differences in stable versus unstable spondylolisthesis.

Over the past decades, there has been an upward trend in the total number of lumbar fusion procedures worldwide. Martin *et al* found a 62.3% increase in elective lumbar fusion surgery between 2004 and 2015. Patients with DS accounted for most elective fusion procedures in the USA (45·2% in 2015) and had the greatest increase (111%).^9^ Analysis of the Norwegian Patient Registry revealed that the rate of complex lumbar procedures (of which spinal fusion accounted for 94%) increased by 154% between 1999 and 2013.^30^ Spinal fusion in properly indicated cases of DS results in excellent long-term (10 years) clinical outcomes.^31^ However, it is a much more invasive and expensive procedure^32^ with a higher incidence of complications than isolated decompression.^12^ It is also associated with the development of degeneration or even symptomatic stenosis or instability of the adjacent spinal segment (ASD, adjacent segment disease). The incidence of ASD following lumbar fusion is 9% with a reoperation rate of 6.2% at 5 years postoperatively.^33^ Our results indicate a higher risk of complications after spinal fusion. Spinal fusion is also associated with extended hospital stay and higher blood loss.

Some published systematic reviews did not find significant differences between the two treatment options.^5 8 11 12 14^ On contrary, other meta-analyses^6 7 13^ found greater efficacy of decompression with fusion than decompression alone. In addition to other limitations, all the reviews inappropriately^15^ combined RCTs with observational studies. Moreover, some studies^7 8 11 13 14^ used data from pseudo-randomised trials in which an old fusion technique (Steffee plates)^34^ or non-instrumented fusion^35^ was used. For our review, we included only RCTs in which the fusion was performed using pedicle screw fixation, which has been established as the gold standard for spinal fusion because of its anchoring strength.^36^ The use of anterior column support by adding interbody fusion does not influence the clinical outcomes compared with DS cases treated by posterolateral fusion.^37^ Therefore, we included both techniques in the analysis. We excluded historical studies comparing cases after non-instrumented fusion due to its lower rate of solid fusion and higher rate of definitive pseudarthrosis.^38^

According to the latest recommendations of The North American Spine Society from 2016 based on older observational data, simple decompression may be considered for symptomatic DS with low-grade (up to 20% anteroposterior caudal vertebral body) slip unresponsive to conservative treatment. The authors noted that in the case of preserving medial structures, it leads to equivalent results as instrumented decompression. Based on the available evidence, it was not possible to make recommendations for or against supplementing fusion in these patients or to predict the success of surgery in terms of age, comorbidities, duration of symptoms or body mass index.^18^ In 2020, Sharif *et al* published the consensus of the World Federation of Neurosurgical Societies Spine Committee on indications for lumbar spine fusion in DS. They recommended isolated decompression for patients with spinal stenosis without signs of instability and predominant leg pain. Lumbar fusion may be added in patients with unstable symptomatic DS or spinal deformity and in those who undergo bilateral facetectomy of over 50%.^39^ Our results support that these recommendations in that fusion are generally no added benefit in DS. However, we could not analyse the outcome according to the level of vertebral slip or instability. Despite the consensus of the professional societies and our findings, the survey studies demonstrate a huge discrepancy among spine surgeons in the surgical management of DS. For instance, Spina *et al* found that 40% of surgeons would always perform fusion in stable DS.^40^ Schroeder *et al* reported that older patients would be more likely treated by isolated decompression. Conversely, instability or low back pain made a fusion more likely than an isolated decompression. At least 2.5% of surgeons in every circumstance recommend isolated decompression, and up to 53% of surgeons recommend this approach in older patients without considerable low back pain or instability.^41^ Despite these recommendations and responses, registry-based studies found that 96% of patients with DS in the USA underwent a fusion in 2011^42^ and 90.6% between 2005 and 2015.^10^ Analysis of data from multicentre studies from Canada (2015–2019)^43^ and France (2009–2013)^44^ showed that fusion was performed in 76% and 83% of DS cases, respectively. In the DS cohort of the Spine Patient Outcomes Research Trial (SPORT), only 7% of patients had instability. Leg pain as the predominant clinical symptom was present in 34% of patients, back pain in 26% and a combination in 40%.^4^ Assuming this cohort represents patients undergoing surgery for DS, one would expect over 4%–24%^10 42–44^ would be a candidate for simple decompression. The high proportion of fusion may be related to no clear evidence for its use, rapid increase in the global instrumentation industry^39^ and insurance status in countries with private insurance. John *et al* reported that private insurance patients had the highest incidence and the highest annual growth of spinal fusion in the USA.^45^

DS can be well demonstrated on a lateral spine X-ray and is often evident on MRI examination. The simple presence of DS does not indicate biomechanical instability, but sagittally oriented facets and substantial facet opening are more predictive of instability.^46^ DS is divided in the literature into static (‘stable’), that is, unchanged in different positions of the spine and dynamic (‘unstable’). Although the difference in slip of at least 3 mm between flexion and extension is most often mentioned in the literature as a sign of instability, there is no clear definition of unstable spondylolisthesis.^40^ A consensus has been reached on the need to fuse an unstable spinal segment, regardless of aetiology.^47^ However, the evidence that ‘instability’ in DS is a treatment effect modifier when comparing decompression with and without fusion is lacking.^39^ On the other hand, it is generally accepted that fusion has no added clinical advantages in treating isolated LSS.^11^ LSS associated with DS makes surgical management more complex and controversial. The resection of the posterior vertebral structures carries a potential risk of developing iatrogenic instability after isolated decompression. This risk is probably comparable to developing ASD after fusion as our results show a similar reoperation rate in both interventions. Evidence shows that symptomatic progression of the slip in patients after decompression without solid fusion becomes apparent only in long-term follow-ups.^48^ However, this evidence is not supported by Försth *et al* 29 with 5 years of follow-up and Inose *et al* 25 with 12 years of follow-up. These authors did not find a difference in the reoperation rate between groups.

This study had some strengths. First, we focused exclusively on the spinal fusion using pedicle screw fixation, which is currently recommended and did not include studies with techniques that are no longer used. Second, we included only RCTs with true randomisation to base our conclusions on highly reliable data. Third, we used the highest standard of methods, including a recent and robust search (May 2022), not pooling different study designs, using data from similar studies, and providing the GRADE assessment. Fourth, we verified and triple checked the accuracy of all data and contacted study authors for missing information. Finally, we used data for minimum of a 2-year follow-up because short-term data have limited impact on decision-making in practice and are difficult to interpret.

However, our study is not without limitations. DS is a heterogeneous pathology and we only assessed it in a single group. Thus, exploring which subpopulations may benefit from adding fusion is needed. We could not provide this information as two trials did not measure stability,^27 28^ and of the other two that measured instability, one did not measure the results of the subgroup^26^ (authors were contacted for this information), and Austevoll *et al* plan to publish data on stability separately.^49^ Kepler *et al* proposed the Clinical And Radiographic Degenerative Spondylolisthesis (CARDS) classification dividing cases of DS based on the presence of disc collapse, instability, focal kyphosis and symptoms.^50^ None of the RCTs evaluated patients in such detail. Despite the not entirely clear definition of the stable/unstable slip, the inclusion of the apparently unstable DS cases with stable ones makes the conclusions of the studies limited. To adopt the best treatment algorithm, future studies should, therefore, adopt CARDS or similar system to determine possible variables affecting treatment results. It means that not only the amount of translation or angular instability from standing to supine (or on flexion-extension views) but also the amount of fluid visible in the facet joints on MRI, the severity of the foraminal stenosis, the orientation of the facet joints, the focal kyphosis at the level of the spondylolisthesis and the dynamic nature of the symptoms from supine to standing should be considered when evaluating patients before treatment.^41^ Moreover, the data might be influenced by the proficiency of the operating surgeons. It was not possible to adjust for this factor in the meta-analysis.

Our systematic review and meta-analysis were designed and carried out by a national panel of experts in spine surgery and evidence synthesis experts. Information specialists specialising in evidence synthesis searching devised the search strategies and conducted the search in nine databases in May 2022 to capture all available published and unpublished data from RCTs. We aimed to include only the highest level evidence focusing on properly randomised trials and solid definitions of DS and fusion technique (pedicle screw fixation). Despite all these efforts, another limitation of this review is the inclusion of only four trials with just over 500 participants. Due to many difficulties associated with conducting neurosurgical RCTs,^51^ the trials are scarce; however, two are currently registered (NCT02348645 and DRKS00000237).

After careful examination of our meta-analysis using the GRADE approach, the certainty in the evidence on the safety and efficacy of isolated decompression was rated as low to moderate. Thus, future research is unlikely to change our confidence in the estimate of effect. Therefore, conclusions of our study should be considered strong enough to influence the clinical practice. Our national guideline development group has formulated a conditional recommendation based on these findings which will be published subsequently. The main limitation of this review is, therefore, the inability to provide more specific findings on who might benefit from adding fusion to decompression, as discussed above, and this decision needs to be based on observational data and clinical expertise in each individual case, until further data are published by Austevoll *et al*.

## Conclusions

Our findings provide clinicians and healthcare policy makers with a comprehensive assessment and high-quality evidence on the safety and efficacy of simple decompression as a superior option for patients with stable DS. This conclusion might be especially useful for patients in higher age groups who are likely to be better served by the lower morbidity associated with decompression alone.

## Data Availability

All data relevant to the study are included in the article or uploaded as supplementary information.
