# Supplementary material for: Decompression alone versus decompression with instrumented fusion in the treatment of lumbar degenerative spondylolisthesis: a systematic review and meta-analysis of randomised trials
Source: J Neurol Neurosurg Psychiatry. 2023 Feb 27;94(8):657–66. doi: 10.1136/jnnp-2022-330158 (PMC10359551; doi:10.1136/jnnp-2022-330158)
Supplement: Supplementary data [file jnnp-2022-330158supp001.pdf]

Supplementary material

Appendix A: Search strategies

Appendix B: Risk of bias assessments (Cochrane RoB 2) for all outcomes used in the review

Appendix C: List of studies excluded at full-text screening stage

Appendix D: Additional meta-analyses

Figure S1: Forest plot for reoperation rate at a minimum of 2-year follow-up

Complication rate (narrative)

Figure S2: Forest plot for length of hospital stay (days)

Figure S3: Forest plot for duration of surgery (mins)

Figure S4: Forest plot for blood loss (ml)

Sensitivity analysis for clustering due to possible surgeon differences

Table: An overview of studies included in meta-analysis for each outcome

Appendix E: PROSPERO search results for underway reviews

Appendix F: Detailed characteristics of included studies

Appendix G: PRISMA checklist for abstract and the main manuscript

Appendix A: Search strategies

MEDLINE(R) ALL <1946 to May 23, 2022> (Ovid)

Search was conducted on 23th May 2022 at 08:57 am (CET).

#	search string	# of results
1	Spondylolisthesis/	5126
2	Lumbosacral Region/ or Lumbar Vertebrae/	69609
3	degenerat*.kf,kw,tw.	238211
4	and/1-3	1399
5	(spondylol#st* or spondyl#st* or anterol#st* or spondyloptos#s or spondylo-l#st* or (vertebra* and (slip* or slid*))).kf,kw,tw.	7031
6	(L1 or L2 or L3 or L4 or L5 or S1 or lumb*).kf,kw,tw.	255199
7	degenerat*.kf,kw,tw.	238211
8	and/5-7	2373
9	4 or 8	2621
10	exp Decompression, Surgical/	34213
11	Foraminotomy/ or Laminoplasty/	861
12	decompress*.kf,kw,tw.	51722

13	(foraminotom* or foraminectom* or lamin?ectom* or hemilamin?ectom* or laminotom* or lamino-tom* or rachi?tom* or rhachi?tom* or spondylectom* or dis#ectom* or facetectom* or corpectom* or vertebr?ectom* or lamin#plast* or lamin#-plast* or spondylotom*).kf,kw,tw.	23622
14	or/10-13	79479
15	exp Arthrodesis/	39195
16	(art?rodes* or artificial ankylos#s or fusion* or spondylodes#s or spondylosyndes#s).kf,kw,tw.	244455
17	or/15-16	254806
18	9 and 14 and 17	820

Embase <1974 to 2022 May 23> (Ovid)

Search was conducted on 23th May 2022 at 9:04 am (CET).

#	search string	# of results
1	exp spondylolisthesis/	9854
2	exp lumbar spine/	75528
3	degenerat*.kf,kw,tw.	305596
4	and/1-3	1574
5	(spondylol#st* or spondyl#st* or anterol#st* or spondyloptos#s or spondylo-l#st* or (vertebra* and (slip* or slid*))).kf,kw,tw.	8802
6	(L1 or L2 or L3 or L4 or L5 or S1 or lumb*).kf,kw,tw.	348780
7	degenerat*.kf,kw,tw.	305596
8	and/5-7	3397
9	4 or 8	3711
10	exp spinal cord surgery/	40888
11	exp foraminotomy/	1050
12	decompress*.kf,kw,tw.	66402
13	(foraminotom* or foraminectom* or lamin?ectom* or hemilamin?ectom* or laminotom* or lamino-tom* or rachi?tom* or	30084

	rhachi?tom* or spondylectom* or dis#ectom* or facetectom* or corpectom* or vertebr?ectom* or lamin#plast* or lamin#-plast* or spondylotom*).kf,kw,tw.	
14	or/10-13	106489
15	exp arthrodesis/	51105
16	(art?rodes* or artificial ankylos#s or fusion* or spondylodes#s or spondylosyndes#s).kf,kw,tw.	300059
17	15 or 16	312182
18	9 and 14 and 17	1231

Emcare <1995 to 2022 Week 20> (Ovid)

Search was conducted on 23th May 2022 at 9:40 am (CET).

#	search string	# of results
1	exp spondylolisthesis/	3030
2	exp lumbar spine/	22940
3	degenerat*.kf,kw,tw.	49061
4	and/1-3	474
5	(spondylol#st* or spondyl#st* or anterol#st* or spondyloptos#s or spondylo-l#st* or (vertebra* and (slip* or slid*))).kf,kw,tw.	2562
6	(L1 or L2 or L3 or L4 or L5 or S1 or lumb*).kf,kw,tw.	70883
7	degenerat*.kf,kw,tw.	49061
8	and/5-7	979
9	4 or 8	1071
10	exp spinal cord surgery/	9755
11	exp foraminotomy/	198
12	decompress*.kf,kw,tw.	13985
13	(foraminotom* or foraminectom* or lamin?ectom* or hemilamin?ectom* or laminotom* or lamino-tom* or rachi?tom* or	7114

	rhachi?tom* or spondylectom* or dis#ectom* or facetectom* or corpectom* or vertebr?ectom* or lamin#plast* or lamin#-plast* or spondylotom*).kf,kw,tw.	
14	or/10-13	23624
15	exp arthrodesis/	18777
16	(art?rodes* or artificial ankylos#s or fusion* or spondylodes#s or spondylosyndes#s).kf,kw,tw.	42100
17	15 or 16	46254
18	9 and 14 and 17	328

Cochrane Library

Search was conducted on 23th May 2022 at 10:25 am (CET).

#	search string	# of results
1	MeSH descriptor: [Spondylolisthesis] this term only	238
2	MeSH descriptor: [Lumbosacral Region] this term only	518
3	MeSH descriptor: [Lumbar Vertebrae] this term only	2816
4	#2 OR #3	3254
5	(degenerat*):ti,ab,kw	10718
6	#1 AND #4 AND #5	81
7	(spondylol?st* or spondyl?st* or anterol?st* or spondyloptos?s or (spondylo NEXT l?st*) or (vertebra* and (slip* or slid*))):ti,ab,kw	866
8	(L1 or L2 or L3 or L4 or L5 or S1 or lumb*):ti,ab,kw	27036
9	(degenerat*):ti,ab,kw	10718
10	#7 AND #8 AND #9	422
11	#6 OR #10	422
12	MeSH descriptor: [Decompression, Surgical] explode all trees	1219
13	MeSH descriptor: [Foraminotomy] this term only	3

14	MeSH descriptor: [Laminoplasty] this term only	14
15	(decompress*):ti,ab,kw	3647
16	(foraminotom* or foraminectom* or lamin?ectom* or hemilamin?ectom* or laminotom* or (lamino NEXT tom*) or rachi?tom or rhachi?tom* or spondylectom* or dis?ectom* or facetectom* or corpectom* or vertebr?ectom* or lamin?plast* or (lamin? NEXT plast*) or spondylotom*):ti,ab,kw	2419
17	14-#16	5604
18	MeSH descriptor: [Arthrodesis] explode all trees	1131
19	(art?rodes* or (artificial NEXT ankylos?s) or fusion* or spondylodes?s or spondylosyndes?s):ti,ab,kw	8364
20	#18 OR #19	8364
21	#11 AND #17 AND #20<br>Cochrane Database of Systematic Reviews, Issue 5 of 12, May 2022<br>Cochrane Central Register of Controlled Trials, Issue 4 of 12, April 2022	1<br>156

CINAHL with Full Text (EBSCO)

Search was conducted on 23th May 2022 at 11:10 am (CET).

#	search string	# of results
1	(MH "Spondylolisthesis")	1623
2	(MH "Lumbar Vertebrae")	19723
3	TI (degenerat*) OR AB (degenerat*)	30760
4	S1 AND S2 AND S3	516
5	TI ((spondylol?st* OR spondyl?st* OR anterol?st* OR spondyloptos?s OR "spondylo-l?st*" OR (vertebra* AND (slip* OR slid*))) OR AB ((spondylol?st* OR spondyl?st* OR anterol?st* OR spondyloptos?s OR "spondylo-l?st*" OR (vertebra* AND (slip* OR slid*))))	2228
6	TI (L1 or L2 or L3 or L4 or L5 or S1 or lumb*) OR AB (L1 or L2 or L3 or L4 or L5 or S1 or lumb*)	41745
7	TI (degenerat*) OR AB (degenerat*)	30760
8	S5 AND S6 AND S7	944
9	S4 OR S8	1003
10	(MH "Decompression, Surgical+")	6121
11	(MH "Laminectomy") OR (MH "Laminoplasty") OR (MH "Diskectomy")	5049
12	TI (decompress*) OR AB (decompress*)	10304

13	TI (foraminotom* or foraminectom* or lamin#ectom* or hemilamin#ectom* or laminotom* or "lamino-tom*" or rachi#tom* or rhachi#tom* or spondylectom* or dis?ectom* or facetectom* or corpectom* or vertebr#ectom* or lamin?plast* or "lamin?-plast*" or spondylotom*) OR AB (foraminotom* or foraminectom* or lamin#ectom* or hemilamin#ectom* or laminotom* or "lamino-tom*" or rachi#tom* or rhachi#tom* or spondylectom* or dis?ectom* or facetectom* or corpectom* or vertebr#ectom* or lamin?plast* or "lamin?-plast*" or spondylotom*)	6194
14	S10 OR S11 OR S12 OR S13	18510
15	(MH "Arthrodesis+")	14324
16	TI (art#rodes* or "artificial ankylos?s" or fusion* or spondylodes?s or spondylosyndes?s) OR AB (art#rodes* or "artificial ankylos?s" or fusion* or spondylodes?s or spondylosyndes?s)	26558
17	S15 OR S16	30320
18	S9 AND S14 AND S17	286

Scopus

Search was conducted on 23th May 2022 at 12:20 pm (CET).

#	search string	# of results
1	TITLE-ABS (spondylol?st* OR spondyl?st* OR anterol?st* OR spondyloptos?s OR "spondylo-l?st*" OR (vertebra* AND (slip* OR slid*))) OR AUTHKEY (spondylol?st* OR spondyl?st* OR anterol?st* OR spondyloptos?s OR "spondylo-l?st*" OR (vertebra* AND (slip* OR slid*)))	8336
2	TITLE-ABS ("l1" OR "l2" OR "l3" OR "l4" OR "l5" OR "s1" OR lumb*) OR AUTHKEY ("l1" OR "l2" OR "l3" OR "l4" OR "l5" OR "s1" OR lumb*)	468054
3	TITLE-ABS (degenerat*) OR AUTHKEY (degenerat*)	381463
4	#1 AND #2 AND #3	2704
5	TITLE-ABS (decompress*) OR AUTHKEY (decompress*)	74694
6	TITLE-ABS (foraminotom* OR foraminectom* OR laminoectom* OR laminectom* OR hemilaminoectom* OR hemilaminectom* OR laminotom* OR "lamino-tom*" OR rachitom* OR rachiotom* OR rhachitom* OR rhachiotom* OR spondylectom* OR dis?ectom* OR facetectom* OR corpectom* OR vertebrectom* OR vertebraectom* OR lamin?plast* OR "lamin?-plast*" OR spondylotom*) OR AUTHKEY (foraminotom* OR foraminectom* OR laminoectom* OR laminectom* OR hemilaminoectom* OR hemilaminectom* OR laminotom* OR "lamino-tom*" OR rachitom* OR rachiotom* OR rhachitom* OR rhachiotom* OR spondylectom* OR dis?ectom* OR facetectom* OR corpectom* OR vertebrectom* OR vertebraectom* OR lamin?plast* OR "lamin?-plast*" OR spondylotom*)	26674
7	#5 OR #6	94619

8	TITLE-ABS (artrodes* OR arthrodes* OR "artificial ankylos?s" OR fusion* OR spondylodes?s OR spondylosyndes?s) OR AUTHKEY (artrodes* OR arthrodes* OR "artificial ankylos?s" OR fusion* OR spondylodes?s OR spondylosyndes?s)	537916
9	#4 AND #7 AND #8	778

ProQuest Dissertations & Theses Global

Search was conducted on 23th May 2022 at 13:05 pm (CET).

#	search string	# of results
**1**	TIABSU(spondylol?st* OR spondyl?st* OR anterol?st* OR spondyloptos?s OR "spondylo-l?st*" OR (vertebra* AND (slip* OR slid*)))	184
**2**	TIABSU(L1 OR L2 OR L3 OR L4 OR L5 OR S1 OR lumb*)	18753
**3**	TIABSU(degenerat*)	20104
**4**	S1 AND S2 AND S3	35
**5**	TIABSU(decompress*)	1697
**6**	TIABSU(foraminotom* OR foraminectom* OR laminoectom* OR laminectom* OR hemilaminectom* OR hemilaminoectom* OR laminotom* OR "lamino-tom*" OR rachitom* OR rachiotom* OR rhachitom* OR rhachiotom* OR spondylectom* OR dis?ectom* OR facetectom* OR corpectom* OR vertebrectom* OR vertebraectom* OR lamin?plast* OR "lamin?-plast*" OR spondylotom*)	267
**7**	S5 OR S6	1919
**8**	TIABSU(artrodes* OR arthrodes* OR "artificial ankylos?s" OR fusion* OR spondylodes?s OR spondylosyndes?s)	36725
**9**	S4 AND S7 AND S8	6

WHO International Clinical Trials Registry Platform (ICTRP)

Search was conducted on 23th May 2022 at 13:20-13:25 pm (CET).

#	search string	# of results
**1**	spondylolisthesis OR spondylolysthesis OR spondylisthesis OR spondylysthesis OR anterolisthesis OR anterolysthesis OR spondyloptosis OR spondylo-listhesis OR spondylo-lysthesis **in Field Title**	
**2**	artrodesis OR arthrodesis OR artificial ankylosis OR fusion OR spondylodesis OR spondylosyndesis **– in Field Intervention**	
**3**	Recruitment status – All	
**4**	1 AND 2 AND 3	33

Search was conducted on 23th May 2022 at 13:30-13:33 pm (CET).

#	search string	# of results
**1**	artrodesis OR arthrodesis OR artificial ankylosis OR fusion OR spondylodesis OR spondylosyndesis **– in Field Title**	
**2**	spondylolisthesis OR spondylolysthesis OR spondylisthesis OR spondylysthesis OR anterolisthesis OR anterolysthesis OR spondyloptosis OR spondylo-listhesis OR spondylo-lysthesis **– in Field Condition**	
**3**	Recruitment status – All	
**4**	1 AND 2 AND 3	141

Search was conducted on 23th May 2022 at 13:25-13:30 pm (CET).

#	search string	# of results
**1**	spondylolist* OR spondylolyst* OR spondylist* OR spondylyst* OR anterolist* OR anterolyst* OR spondyloptos* OR spondylo-list* OR spondylo-lyst* **in Field Title**	
**2**	artrodes* OR arthrodes* OR artificial ankylos* OR fusion* OR spondylodes* OR spondylosyndes* **– in Field Condition**	
**3**	Recruitment status – All	
**4**	1 AND 2 AND 3	9

Search was conducted on 23th May 2022 at 13:40–13:45 pm (CET).

#	search string	# of results
**1**	spondylolisthesis OR spondylolysthesis OR spondylisthesis OR spondylysthesis OR anterolisthesis OR anterolysthesis OR spondyloptosis OR spondylo-listhesis OR spondylo-lysthesis **in Field Condition**	
**2**	artrodesis OR arthrodesis OR artificial ankylosis OR fusion OR spondylodesis OR spondylosyndesis **– in Field Intervention**	
**3**	Recruitment status – All	
**4**	1 AND 2 AND 3	68

ClinicalTrials.gov

Search was conducted on 23th May 2022 at 13:55–14:00 pm (CET).

#	search string	# of results
**1**	spondylolisthesis OR spondylolysthesis OR spondylisthesis OR spondylysthesis OR anterolisthesis OR anterolysthesis OR spondyloptosis OR "spondylo-listhesis" OR "spondylo-lysthesis" – **in Field Condition or disease**	
**2**	artrodesis OR arthrodesis OR "artificial ankylosis" OR fusion OR spondylodesis OR spondylosyndesis – **in Field Other terms**	
**3**	1 AND 2	163

Search was conducted on 23th May 2022 at 13:55–14:00 pm (CET).

#	search string	# of results
**1**	artrodesis OR arthrodesis OR "artificial ankylosis" OR fusion OR spondylodesis OR spondylosyndesis – **in Field Condition or disease**	
**2**	spondylolisthesis OR spondylolysthesis OR spondylisthesis OR spondylysthesis OR anterolisthesis OR anterolysthesis OR spondyloptosis OR "spondylo-listhesis" OR "spondylo-lysthesis" – **in Field Other terms**	
**3**	1 AND 2	146

Search was conducted on 23th May 2022 at 14:00–14:05 pm (CET).

#	search string	# of results
**1**	artrodesis OR arthrodesis OR "artificial ankylosis" OR fusion OR spondylodesis OR spondylosyndesis – **in Field Condition or disease**	

**2**	Spondylolisthesis OR spondylolysthesis OR spondylisthesis OR spondylysthesis OR anterolisthesis OR anterolysthesis OR spondyloptosis OR "spondylo-listhesis" OR "spondylo-lysthesis" – **in Field Title**	
**3**	1 AND 2	22

Search was conducted on 23th May 2022 at 14:00–14:05 pm (CET).

#	search string	# of results
**1**	Spondylolisthesis OR spondylolysthesis OR spondylisthesis OR spondylysthesis OR anterolisthesis OR anterolysthesis OR spondyloptosis OR "spondylo-listhesis" OR "spondylo-lysthesis" – **in Field Title**	
**2**	artrodesis OR arthrodesis OR "artificial ankylosis" OR fusion OR spondylodesis OR spondylosyndesis – **in Field Other terms**	
**3**	1 AND 2	31

Search was conducted on 23th May 2022 at 14:00–14:05 pm (CET).

#	search string	# of results
**1**	Spondylolisthesis OR spondylolysthesis OR spondylisthesis OR spondylysthesis OR anterolisthesis OR anterolysthesis OR spondyloptosis OR "spondylo-listhesis" OR "spondylo-lysthesis" – **in Field Other Terms**	
**2**	artrodesis OR arthrodesis OR "artificial ankylosis" OR fusion OR spondylodesis OR spondylosyndesis – **in Field I*ntervention***	
**3**	1 AND 2	163

Search was conducted on 23th May 2022 at 14:05–14:10 pm (CET).

#	search string	# of results
**1**	Spondylolisthesis OR spondylolysthesis OR spondylisthesis OR spondylysthesis OR anterolisthesis OR anterolysthesis OR spondyloptosis OR "spondylo-listhesis" OR "spondylo-lysthesis" – **in Field Other Terms**	
**2**	artrodesis OR arthrodesis OR "artificial ankylosis" OR fusion OR spondylodesis OR spondylosyndesis – **in Field Title**	
**3**	1 AND 2	132

Appendix B: Risk of bias assessment

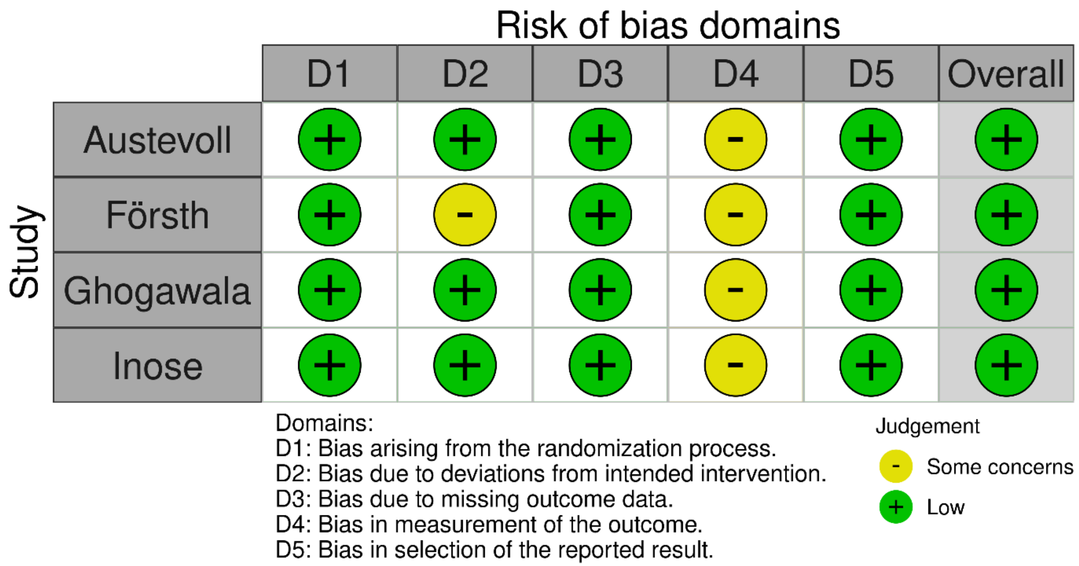

Appendix C: List of studies excluded at full-text screening stage

**Aihara (2 reports)**		Non-randomized
**Bridwell**	USA	pseudo-randomization
**Grob**	Sweden	pseudo-randomization (randomization according to admission date); inappropriate definition of instability; older fusion technique
**Hallett**		Not spondylolisthesis – wrong population
**Herkowitz**	USA	Non-randomized; fusion not instrumented
**Hussanin**	Egypt	Duplicate text to Inose (seems like plagiarism)
**Kleinstueck**		Non-randomized
**Louie**	USA	Commentary – wrong design
**Truszczyńska - Cabak**	Poland	Not spondylolisthesis – wrong population
**White**		Wrong population – disc herniation

Aihara T, Endo K, Sawaji Y, et al. Five-year Reoperation Rates and Causes for Reoperations Following Lumbar Microendoscopic Discectomy and Decompression. Spine 2020; 45(1): 71-7.

Aihara T, Toyone T, Murata Y, Inage K, Urushibara M, Ouchi J. Degenerative Lumbar Spondylolisthesis with Spinal Stenosis: A Comparative Study of 5-Year Outcomes Following Decompression with Fusion and Microendoscopic Decompression. Asian Spine Journal 2018; 12(1): 132-9.

Bridwell KH, Sedgewick TA, O'Brien MF, Lenke LG, Baldus C. The role of fusion and instrumentation in the treatment of degenerative spondylolisthesis with spinal stenosis. *Journal of spinal disorders* 1993; **6**(6): 461-72.

Grob D, Humke T, Dvorak J. Degenerative lumbar spinal stenosis. Decompression with and without arthrodesis. *J Bone Joint Surg Am* 1995; **77**(7): 1036-41.

Hallett A, Huntley JS, Gibson JN. Foraminal stenosis and single-level degenerative disc disease: a randomized controlled trial comparing decompression with decompression and instrumented fusion. Spine (Phila Pa 1976) 2007; 32(13): 1375-80.

Herkowitz HN, Kurz LT. Degenerative lumbar spondylolisthesis with spinal stenosis. A prospective study comparing decompression with decompression and intertransverse process arthrodesis. *J Bone Joint Surg Am* 1991; **73**(6): 802-8.

Hussanin AAAS. Comparison of decompression and decompression plus fusion, for degenerative spondylolisthesis management: Randomized controlled trial. *Egypt J Hosp Med* 2020; **80**(1): 683-7.

Kleinstueck FS, Fekete TF, Mannion AF, et al. To fuse or not to fuse in lumbar degenerative spondylolisthesis: do baseline symptoms help provide the answer? Eur Spine J 2012; 21(2): 268-75.

Louie PK. In Spinal Stenosis with Degenerative Spondylolisthesis, Decompression Surgery Alone Was Noninferior to Decompression Surgery with Instrumented Fusion for Reducing Impairment at 2 Years. J Bone Joint Surg Am 2022; 104(10): 943.

Truszczyńska A, Rąpała K, Łukawski S, et al. Evaluation of functional outcomes in individuals 10 years after posterior lumbar interbody fusion with corundum implants and decompression: a comparison of 2 surgical techniques. *Med Sci Monit* 2014; **20**: 1400-6.

White AH, von Rogov P, Zucherman J, Heiden D. Lumbar laminectomy for herniated disc: a prospective controlled comparison with internal fixation fusion. Spine (Phila Pa 1976) 1987; 12(3): 305-7.

Underway registered trials

Decompression versus Decompression and Fusion in patients with degenerative Spondylolisthesis. A prospective randomized, controlled clinical study. https://trialsearchwhoint/Trial2aspx?TrialID=DRKS00000237 2009. ID: DRKS00000237

Decompression vs. Fusion for Stable Degenerative Spondylolisthesis. https://clinicaltrialsgov/show/NCT02348645 2014. ID: NCT02348645

Appendix D: Additional meta-analyses

Figure S1: Forest plot for reoperation rate at a minimum of 2-year follow-up

	Decompression		Decompression wtih fusion			Odds Ratio
Study or Subgroup	Events	Total	Events	Total	Weight	M-H, Random, 95% CI
Austevoll 2021	15	120	11	121	47.9%	1.43 [0.63, 3.25]
Forsth 2016 (1)	15	68	14	67	48.0%	1.07 [0.47, 2.44]
Inose 2018 (2)	1	29	1	31	4.1%	1.07 [0.06, 17.96]
**Total (95% CI)**		**217**		**219**	**100.0%**	**1.23 [0.70, 2.17]**
Total events	31		26			

Heterogeneity: Tau² = 0.00; Chi² = 0.24, df = 2 (P = 0.88); I² = 0%
Test for overall effect: Z = 0.71 (P = 0.48)

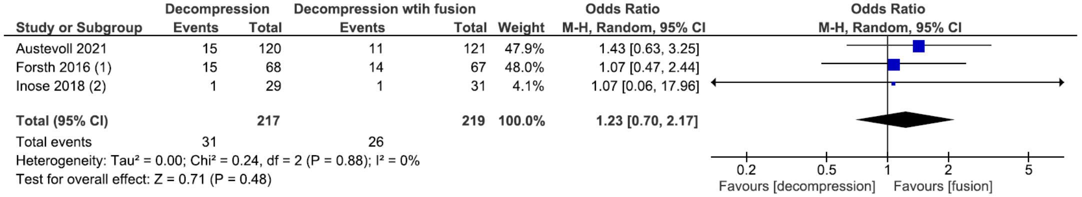

Footnotes
(1) The fusion group might have included up to 5 patients with non-instrumental fusion.
(2) 5-year FU

Complication rate (narrative)

Because the complications were recorded differently in each trial, we decided not to pool the results in a meta-analysis and provide the following summary:

- **Ghogawala et al**. describes perioperative complications within 30 days: The complications in the decompression-alone group included wound infection (1) and new neurologic deficit (1). The complication in the fusion group was pneumonia (1). Minor complications were not recorded. **In total, there were 2 major perioperative complications in the decompression only group and 1 in the fusion group.**
- **Inose et al.** recorded intraoperative and perioperative complications: In the fusion group, the following occurred: dural tears (2), meralgia paresthetica due to compression of the lateral femoral cutaneous nerve (5), pulmonary embolism (1). In the decompression only group a postoperative symptomatic hematoma that required bed rest, but not reoperation, occurred in 1 patient. **In total, there were 8 perioperative complications in the fusion group and 1 in the decompression only group.**
- **Forsth et al.** did not specify the number of complications in the spondylolisthesis subset of the patients. For the combined group of patients (patients with lumbar spinal stenosis with and without degenerative spondylolisthesis) the complication rate was comparable in the decompression only (total 23/124 initially randomized) and the fusion (total 26/123 initially randomized) groups.
- **Austevoll et al.** provides a detailed list of the complications, their severity and approximate time of occurrence. The perioperative complications (up to 3 months after discharge) were: incidental dural tear (7 in D and 17 in F group), surgery on the wrong side or level (1 in each group), hematoma resulting in reoperation during hospital stay (1 in each group), wound infection after discharge (3 in D and 6 in F group), cardiovascular complications (4 in D and 0 in F group), urologic complications (6 in D and 11 in F group), respiratory complications (1 in D and 2 in F group), neurologic deterioration during hospital stay and within 3 months after discharge (4 in D and 9 in F group). **In total, there were 27 complications in the decompression only group and 47 in the fusion group. There were 17 (D) and 29 (F) complications in the respective groups that occurred during the hospital stay.**

- **The combined result for all three trials (Ghogawala, Inose and Austevoll) are: 30 complications in the decompression only group and 56 complications in the fusion group, with data driven mainly by Austevoll et al. due to the highest number of recorded complications. When counting only the complications that occurred during hospital stay in Austevoll trial, the total number of complications in the 3 trials is 20 and 38.**

Figure S2: Forest plot for length of hospital stay (days)

	Decompression			Decompression with fusion				Mean Difference
Study or Subgroup	Mean	SD	Total	Mean	SD	Total	Weight	IV, Random, 95% CI
Austevoll 2021	3.3	0.2	133	5	0.2	129	98.7%	-1.70 [-1.75, -1.65]
Ghogawala 2016	2.6	0.9	35	4.2	0.9	31	1.2%	-1.60 [-2.04, -1.16]
Inose 2018	11.6	2.5	29	14.1	3.6	31	0.1%	-2.50 [-4.06, -0.94]
**Total (95% CI)**			**197**			**191**	**100.0%**	**-1.70 [-1.75, -1.65]**

Heterogeneity: Tau² = 0.00; Chi² = 1.21, df = 2 (P = 0.55); I² = 0%
Test for overall effect: Z = 69.22 (P < 0.00001)

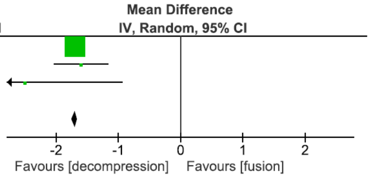

Figure S3: Forest plot for duration of surgery (mins)

	Decompression			Decompression with fusion				Mean Difference
Study or Subgroup	Mean	SD	Total	Mean	SD	Total	Weight	IV, Random, 95% CI
Austevoll 2021 (1)	104	57.8029	133	174	57.8029	129	25.8%	-70.00 [-84.00, -56.00]
Forsth 2016 (2)	95	40	66	149	44	67	25.8%	-54.00 [-68.29, -39.71]
Ghogawala 2016	124.4	34.2	35	289.6	66.3	31	24.0%	-165.20 [-191.14, -139.26]
Inose 2018	148	46	29	244	50	31	24.3%	-96.00 [-120.29, -71.71]
**Total (95% CI)**			**263**			**258**	**100.0%**	**-95.06 [-135.29, -54.82]**

Heterogeneity: Tau² = 1579.17; Chi² = 57.60, df = 3 (P < 0.00001); I² = 95%
Test for overall effect: Z = 4.63 (P < 0.00001)

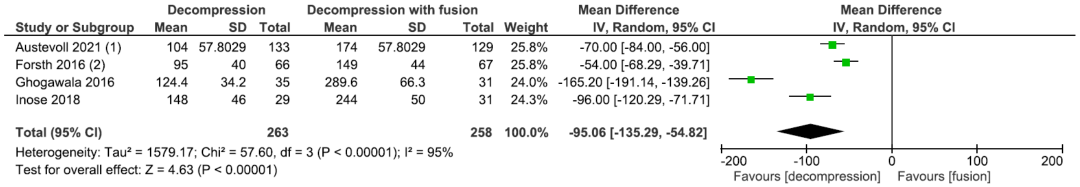

Footnotes
(1) SD calculated from 95% CI
(2) The fusion group might have included up to 5 patients with non-instrumental fusion.

Figure S4: Forest plot for blood loss (ml)

Study or Subgroup	Decompression Mean	SD	Total	Decompression with fusion Mean	SD	Total	Weight	Mean Difference IV, Random, 95% CI
Austevoll 2021	141	134	133	429	278	129	34.9%	-288.00 [-341.10, -234.90]
Forsth 2016 (1)	311	314	66	686	434	67	17.4%	-375.00 [-503.60, -246.40]
Ghogawala 2016	83.4	63.5	35	513.7	334.4	31	19.0%	-430.30 [-549.88, -310.72]
Inose 2018	80.3	62.5	29	334.8	206.3	31	28.7%	-254.50 [-330.60, -178.40]
**Total (95% CI)**			**263**			**258**	**100.0%**	**-320.55 [-389.61, -251.49]**

Heterogeneity: Tau² = 2821.13; Chi² = 7.42, df = 3 (P = 0.06); I² = 60%
Test for overall effect: Z = 9.10 (P < 0.00001)

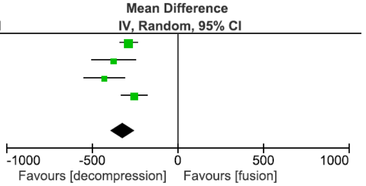

Footnotes
(1) The fusion group might have included up to 5 patients with non-instrumental fusion.

Table: Studies used to calculate effect estimate for each outcome (× means the study was used)

	Austevoll 2021	Försth 2016	Ghogawala 2016	Inose 2018
**ODI**	×	×	×	
**Back pain**	×	×		×
**Leg pain**	×	×		×
**Reoperation rate**	×	×		×
**Complication rate (narrative)**	×		×	×
**Hospital stay**	×		×	×
**Duration of surgery**	×	×	×	×
**Blood loss**	×	×	×	×

Appendix E: PROSPERO search results for underway reviews

Manon Dijkerman, Carmen Vleggeert-Lankamp, Wilco Jacobs, Gijs Overdevest. Comparing the outcome of decompression with and without fusion between patients suffering from degenerative spondylolisthesis in combination with lumbar stenosis. PROSPERO 2015 CRD42015019887 Available from: https://www.crd.york.ac.uk/prospero/display_record.php?ID=CRD42015019887

Published: http://dx.doi.org/10.1007/s00586-017-5436-5

Scott Koenig, Julio Jauregui. Decompression vs. fusion for grade 1 degenerative spondylolisthesis: a meta-analysis. PROSPERO 2017 CRD42017057587 Available from: https://www.crd.york.ac.uk/prospero/display_record.php?ID=CRD42017057587

Published: http://dx.doi.org/10.1177/2192568218777476

Haifeng Liang. Systematic review and meta-analysis of decompression alone versus decompression plus fusion for lumbar spondylolisthesis. PROSPERO 2017 CRD42017055598 Available from: https://www.crd.york.ac.uk/prospero/display_record.php?ID=CRD42017055598

Published: http://dx.doi.org/10.1007/s00586-017-5200-x

Raymond Pranata, Michael Lim, Rachel Vania. Decompression Alone Compared to Decompression Plus Fusion in Patients with Lumbar Spondylolisthesis: Systematic Review, Meta-analysis, and Meta-regression. PROSPERO 2020 CRD42020211904 Available from: https://www.crd.york.ac.uk/prospero/display_record.php?ID=CRD42020211904

Published: http://dx.doi.org/10.14444/8179

Fei-Long Wei, Xiao-Dong Yan. Decompression with or without Fusion in Degenerative Lumbar Spondylolisthesis. PROSPERO 2022 CRD42022310645 Available from: https://www.crd.york.ac.uk/prospero/display_record.php?ID=CRD42022310645

Registered after our protocol

Appendix F: Detailed characteristics of included studies

Author, year<br><br>Country	Inclusion criteria	Exclusion criteria	Missing data	Primary outcomes	Adverse/safety events	Objective of the study	Ethnicity	Relevant concomitant treatments	N. of treatment arms	Risk of bias domains	Power calculation

**Austevoll, 2021**<br><br>**NORDSTEN-DS trial**<br><br>Norway	Eligible patients were 18 to 80 years of age with neurogenic claudication or radicular radiating pain in the lower limbs that had not responded to at least 3 months of conservative care. Patients had to have radiographic evidence of spinal stenosis verified by magnetic resonance imaging (MRI) and have degenerative spondylolisthesis solely at the stenotic level of at least 3 mm verified by standing plain radiographs obtained in the lateral view. Patients were included regardless of the grade of slippage above 3 mm and regardless of the result of the flexion–extension	a score of less than 25 on the Oswestry Disability Index (ODI) was an exclusion criterion	Multiple imputation was performed if data were missing at baseline (4 patients) or at the 2-year follow-up (22 patients).	A reduction of at least 30% in the score on the Oswestry Disability Index (ODI; range, 0 to 100, with higher scores indicating more impairment) during the 2 years after surgery, with a noninferiority margin of −15 percentage points.	The complications recorded by Austevoll et al. were: The perioperative complications (up to 3 months after discharge) were: incidental dural tear (7 in D and 17 in F group), surgery on the wrong side or level (1 in each group), hematoma resulting in reoperation during hospital stay (1 in each group), wound infection after discharge (3 in D and 6 in F group), cardiovascular complications (4 in D and 0 in F group), urologic complications (6 in D and 11 in F group), respiratory complications (1 in D and 2	"decompression surgery alone is noninferior to decompression with instrumented fusion." " we consider the conclusion of noninferiority to be valid."<br><br>**Interim analysis:** When 150 of the trial patients had completed their 1-year assessment, a third-party statistician, who was unaware of the treatment-group assignments, performed an interim analysis for safety and efficacy according to the protocol. Permission to continue the trial, and no other results,	NR	NR	2 arms	True randomisation of 1:1 ratio<br><br>Allocation: computer-generated, stratified according to centre in blocks of 4-6<br><br>Blinding: Patients not blinded; Investigators (outcome assessment) blinded<br><br>Modified intention-to-treat analysis: all patients who received the trial treatment in accordance with the randomization with available data (with imputations for missing data)	Done and provided in the trial report

	radiographs to detect dynamic slippage of vertebral bodies.				in F group), neurologic deterioration during hospital stay and within 3 months after discharge (4 in D and 9 in F group).	was disclosed to the research group on February 28, 2017.						

**Försth, 2016**<br><br>Sweden	Pseudoclaudication in one or both legs and back pain (score on visual-analogue scale >30)* (VAS 0-100)<br>1 or 2 adjacent stenotic segments (cross-section area of the dural sac ≤75 mm2) between L2 and the sacrum on magnetic resonance imaging<br>Duration of symptoms >6 mo<br>Written informed consent	Spondylolysis<br>Degenerative lumbar scoliosis (Cobb angle >20 degrees)<br>History of lumbar spinal surgery for spinal stenosis or instability<br>Stenosis not caused by degenerative changes<br>Stenosis caused by a herniated disk<br>Other specific spinal conditions (e.g., ankylosing spondylitis, cancer, or neurologic disorders)<br>History of vertebral compression fractures in affected segments<br>Psychological disorders (e.g., dementia or drug abuse) that caused the surgeon to consider participation to	We used multiple imputation to create five estimates of missing data in the health economic evaluation, including values for age, sex, and scores on the visual-analogue scales for back pain and leg pain, the ODI, and the EQ-5D. Values for the health economic evaluation were imputed for 30% of patients at the 6-month follow-up, 33% at the 1-year follow-up, and 14% at the 2-	The primary outcome was the score on the Oswestry Disability Index (ODI; which ranges from 0 to 100, with higher scores indicating more severe disability) 2 years after surgery	Myocardial infarction, stroke, or thromboembolic events occurred in 3 patients (3%) in the fusion group and in 5 patients (4%) in the decompression-alone group. (The data are not available for the subsets of spondylolisthesis patients.)	" clinical superiority trial"<br>" the lack of superiority of decompression plus fusion seemed to persist at 5 years among those patients."	NR	NR	2x2 arms (4 strata: decompression only without spondylolisthesis and with spondylolisthesis, decompression with fusion with and without spondylolisthesis)<br>Only spondylolisthesis patients used in this review	True randomisation of 1:1 ratio<br><br>Allocation: computer-generated, stratified according to the presence/absence of degenerative spondylolisthesis<br><br>Blinding: Patients not blinded; Investigators not blinded<br><br>Primary analysis: per-protocol (patients who underwent the assigned surgery and completed the 2-year followup); (intention-to-treat analysis was not available for the two included arms in this review)	Done and provided in the trial report

		be inappropriate	year follow-up. Calculations of standard deviation and error were adjusted to account for the increased size of the data set								

**Ghogawala, 2016**<br><br>**SLIP trial**<br><br>USA	grade I lumbar spondylolisthesis (degree of spondylolisthesis, 3 to 14 mm) with lumbar stenosis and neurogenic claudication with or without lumbar radiculopathy	Radiography revealed lumbar instability (motion of >3 mm at the level of listhesis, as measured on flexion–extension radiographs of the lumbar spine)<br>Judged by the enrolling surgeon to have lumbar instability because of a history of mechanical low back pain with axial loading of the spine<br>Previous lumbar spinal surgery<br>American Society of Anesthesiologists (ASA) class IV or higher disease (with classes ranging from I to VI and higher classes indicating more severe	NR	The primary outcome measure was the change in the physical-component summary score of the Medical Outcomes Study 36-Item Short-Form Health Survey (SF-36; range, 0 to 100, with higher scores indicating better quality of life) 2 years after surgery.	Ghogawala et al. only recorded major complications. “The complications in the decompression-alone group included wound infection and new neurologic deficit. The complication in the fusion group was pneumonia. All complications were identified within 30 days. Minor complications were not recorded.”	NR	NR	NR	2 arms	True randomisation<br><br>Allocation: Unknown<br><br>Blinding: Patients not blinded; Investigators not blinded<br><br>Modified intention-to-treat analysis: all patients who had follow-up assessments, according to their original randomized treatment assignments	Done and provided in the trial report

		systemic disease)									

**Inose, 2018**<br><br>Japan	NR	Patients over the age of 75 years at the time of the enrolment were excluded. Patients with a previous history of lumbar spinal surgery, multilevel stenosis, or foraminal stenosis were also excluded	NR	The change in VAS for lower back pain	The following was observed: dural tear, delusion, hematoma, meralgia, pulmonary embolism, misplacement of pedicle screw (It is not clear whether these are for 1- or 5-year follow-up or less. Inose states these are related to “intraoperative and perioperative complications”.)	NR	NR	NR	3 arms (decompression alone (decompression group), decompression and posterolateral fusion with autogenous iliac bone graft and pedicle screw fixation (fusion group), or decompression plus stabilization using the Graf system (stabilization group using a braided polypropylene tension band to link the titanium pedicle screws)	True randomisation of 1:1:1 ratio<br><br>Allocation: computer-generated<br><br>Blinding: Patients not blinded; Investigators not blinded<br><br>Primary analysis: per-protocol	Done and provided in the trial report

Appendix G: PRISMA checklist for abstract and the main manuscript

PRISMA 2020 for Abstracts Checklist

Section and Topic	Item #	Checklist item	Reported (Yes/No)
**TITLE**			
Title	1	Identify the report as a systematic review.	Y
**BACKGROUND**			
Objectives	2	Provide an explicit statement of the main objective(s) or question(s) the review addresses.	Y
**METHODS**			
Eligibility criteria	3	Specify the inclusion and exclusion criteria for the review.	N (not enough space to mention all details, defined as part of the title, background and the first part of methods)
Information sources	4	Specify the information sources (e.g. databases, registers) used to identify studies and the date when each was last searched.	N (not enough space, we mentioned the number of databases and the date)
Risk of bias	5	Specify the methods used to assess risk of bias in the included studies.	Y (tool not mentioned

Section and Topic	Item #	Checklist item	Reported (Yes/No)
			due to lack of space)
Synthesis of results	6	Specify the methods used to present and synthesise results.	Y
**RESULTS**			
Included studies	7	Give the total number of included studies and participants and summarise relevant characteristics of studies.	Y
Synthesis of results	8	Present results for main outcomes, preferably indicating the number of included studies and participants for each. If meta-analysis was done, report the summary estimate and confidence/credible interval. If comparing groups, indicate the direction of the effect (i.e. which group is favoured).	Y
**DISCUSSION**			
Limitations of evidence	9	Provide a brief summary of the limitations of the evidence included in the review (e.g. study risk of bias, inconsistency and imprecision).	N (not enough space, this is provided in the full manuscript, and we provide certainty in the evidence assessments that are more informative than risk of bias itself)
Interpretation	10	Provide a general interpretation of the results and important implications.	Y
**OTHER**			
Funding	11	Specify the primary source of funding for the review.	Y
Registration	12	Provide the register name and registration number.	Y (database

Section and Topic	Item #	Checklist item	Reported (Yes/No)
			and number provided)

From: Page MJ, McKenzie JE, Bossuyt PM, Boutron I, Hoffmann TC, Mulrow CD, et al. The PRISMA 2020 statement: an updated guideline for reporting systematic reviews. BMJ 2021;372:n71. doi: 10.1136/bmj.n71

For more information, visit: http://www.prisma-statement.org/

PRISMA 2020 Checklist

Section and Topic	Item #	Checklist item	Location where item is reported
**TITLE**			
Title	1	Identify the report as a systematic review.	Page 1
**ABSTRACT**			
Abstract	2	See the PRISMA 2020 for Abstracts checklist.	Supplement
**INTRODUCTION**			
Rationale	3	Describe the rationale for the review in the context of existing knowledge.	Page 2
Objectives	4	Provide an explicit statement of the objective(s) or question(s) the review addresses.	Page 2
**METHODS**			
Eligibility criteria	5	Specify the inclusion and exclusion criteria for the review and how studies were grouped for the syntheses.	Page 3
Information sources	6	Specify all databases, registers, websites, organisations, reference lists and other sources searched or consulted to identify studies. Specify the date when each source was last searched or consulted.	Page 3
Search strategy	7	Present the full search strategies for all databases, registers and websites, including any filters and limits used.	Supplement
Selection process	8	Specify the methods used to decide whether a study met the inclusion criteria of the review, including how many reviewers screened each record and each report retrieved, whether they worked independently, and if applicable, details of automation tools used in the process.	Page 3

Section and Topic	Item #	Checklist item	Location where item is reported
Data collection process	9	Specify the methods used to collect data from reports, including how many reviewers collected data from each report, whether they worked independently, any processes for obtaining or confirming data from study investigators, and if applicable, details of automation tools used in the process.	Page 3
Data items	10a	List and define all outcomes for which data were sought. Specify whether all results that were compatible with each outcome domain in each study were sought (e.g. for all measures, time points, analyses), and if not, the methods used to decide which results to collect.	Page 3
	10b	List and define all other variables for which data were sought (e.g. participant and intervention characteristics, funding sources). Describe any assumptions made about any missing or unclear information.	Page 4
Study risk of bias assessment	11	Specify the methods used to assess risk of bias in the included studies, including details of the tool(s) used, how many reviewers assessed each study and whether they worked independently, and if applicable, details of automation tools used in the process.	Page 4
Effect measures	12	Specify for each outcome the effect measure(s) (e.g. risk ratio, mean difference) used in the synthesis or presentation of results.	Page 4, Supplement data method
Synthesis methods	13a	Describe the processes used to decide which studies were eligible for each synthesis (e.g. tabulating the study intervention characteristics and comparing against the planned groups for each synthesis (item #5)).	Page 4, Supplement data method
	13b	Describe any methods required to prepare the data for presentation or synthesis, such as handling of missing summary statistics, or data conversions.	Page 4, Supplement data method
	13c	Describe any methods used to tabulate or visually display results of individual studies and syntheses.	Page 4, Supplement data method, also see Appendix D
	13d	Describe any methods used to synthesize results and provide a rationale for the choice(s). If meta-analysis was performed, describe the model(s), method(s) to identify the presence and extent of statistical heterogeneity, and software package(s) used.	Page 4, Supplement data method, and Appendix D
	13e	Describe any methods used to explore possible causes of heterogeneity among study results (e.g. subgroup analysis, meta-regression).	Page 4, Supplement data

Section and Topic	Item #	Checklist item	Location where item is reported
			method, also see Appendix D
	13f	Describe any sensitivity analyses conducted to assess robustness of the synthesized results.	Page 4, Supplement data method, also see Appendix D
Reporting bias assessment	14	Describe any methods used to assess risk of bias due to missing results in a synthesis (arising from reporting biases).	Page 4, Risk of bias assessment in Appendix B
Certainty assessment	15	Describe any methods used to assess certainty (or confidence) in the body of evidence for an outcome.	Page 4, Supplement data method
**RESULTS**			
Study selection	16a	Describe the results of the search and selection process, from the number of records identified in the search to the number of studies included in the review, ideally using a flow diagram.	Page 5, Figure 1
	16b	Cite studies that might appear to meet the inclusion criteria, but which were excluded, and explain why they were excluded.	Figure 1, Appendix C
Study characteristics	17	Cite each included study and present its characteristics.	Page 5, Table 1, and Appendix F
Risk of bias in studies	18	Present assessments of risk of bias for each included study.	Appendix B
Results of individual studies	19	For all outcomes, present, for each study: (a) summary statistics for each group (where appropriate) and (b) an effect estimate and its precision (e.g. confidence/credible interval), ideally using structured tables or plots.	Page 5, Figures 2-4, Appendix D
Results of syntheses	20a	For each synthesis, briefly summarise the characteristics and risk of bias among contributing studies.	Page 5, Appendix B, and Table 2

Section and Topic	Item #	Checklist item	Location where item is reported
	20b	Present results of all statistical syntheses conducted. If meta-analysis was done, present for each the summary estimate and its precision (e.g. confidence/credible interval) and measures of statistical heterogeneity. If comparing groups, describe the direction of the effect.	Page 5, Figures 2-4, Appendix D
	20c	Present results of all investigations of possible causes of heterogeneity among study results.	Page 5, Appendix D, Table 2
	20d	Present results of all sensitivity analyses conducted to assess the robustness of the synthesized results.	Page 5, Appendix D
Reporting biases	21	Present assessments of risk of bias due to missing results (arising from reporting biases) for each synthesis assessed.	Page 6, Appendix B
Certainty of evidence	22	Present assessments of certainty (or confidence) in the body of evidence for each outcome assessed.	Page 6. Table 2
**DISCUSSION**			
Discussion	23a	Provide a general interpretation of the results in the context of other evidence.	Pages 6–8
	23b	Discuss any limitations of the evidence included in the review.	Pages 8–9
	23c	Discuss any limitations of the review processes used.	Page 8
	23d	Discuss implications of the results for practice, policy, and future research.	Pages 9–10
**OTHER INFORMATION**			
Registration and protocol	24a	Provide registration information for the review, including register name and registration number, or state that the review was not registered.	Page 1 and 3
	24b	Indicate where the review protocol can be accessed, or state that a protocol was not prepared.	Page 1 and 3
	24c	Describe and explain any amendments to information provided at registration or in the protocol.	NA (no changes made to the protocol, protocol was followed)
Support	25	Describe sources of financial or non-financial support for the review, and the role of the funders or sponsors in the review.	Page 1, 4 and 10
Competing interests	26	Declare any competing interests of review authors.	Page 10

Section and Topic	Item #	Checklist item	Location where item is reported
Availability of data, code and other materials	27	Report which of the following are publicly available and where they can be found: template data collection forms; data extracted from included studies; data used for all analyses; analytic code; any other materials used in the review.	Page 10

From: Page MJ, McKenzie JE, Bossuyt PM, Boutron I, Hoffmann TC, Mulrow CD, et al. The PRISMA 2020 statement: an updated guideline for reporting systematic reviews. BMJ 2021;372:n71. doi: 10.1136/bmj.n71

For more information, visit: http://www.prisma-statement.org/
